# Zebrafish models of the immune response: taking it on the ChIn

**DOI:** 10.1186/1741-7007-8-148

**Published:** 2010-12-22

**Authors:** Stephen A Renshaw, Philip W Ingham

**Affiliations:** 1 MRC Centre for Developmental and Biomedical Genetics, University of Sheffield, Western Bank, Sheffield, S10 2TN, UK; 2 Institute of Molecular and Cellular Biology, 61 Biopolis Drive, Proteos, Singapore 138673

## Abstract

The zebrafish is proving to be an extremely versatile new experimental model for unraveling the mysteries of innate immunity and has considerable promise as a system for the identification of novel modulators of this crucial biological process. A rate-limiting factor, however, is the mechanical stimulus required to induce the inflammatory response. A new chemically induced inflammation assay ('ChIn' assay) published in *BMC Biology *obviates this requirement and seems set to accelerate progress in the field.

## Commentary

The rise of multicellular animals, to what we might consider the pinnacle of human culture, relies on their ability to defend themselves against unicellular organisms competing for the same environmental resources. To aid in the constant battle waged with would-be pathogens, powerful and complex immune structures have developed, built on a series of molecular and cellular advances made by evolution hundreds of millions of years ago. Over the past century, an increasing understanding of the immune system, together with advances in public health and antimicrobial chemotherapy, have had a huge impact in preventing infectious disease and extending human lifespan. In recent decades, however, the emergence of multi-drug-resistant bacteria and the inexorable rise in inflammatory diseases threaten to undermine these improvements in health: the need for a detailed understanding of the immune system has never been more pressing. In *BMC Biology*, d'Alençon and colleagues [1] report a novel method for high-throughput *in vivo *analysis of immune-cell function that offers new and exciting prospects for our understanding of the immune system, as well as for the discovery of new drugs with which to manipulate it.

Our understanding of innate immunity began with the observations of Elie Metchnikoff, who in 1882 pricked a starfish larva with a thorn from his garden. The insult provided an immune stimulus comprising both infection and tissue injury, prompting the recruitment of cells that attempted to ingest the thorn. The transparency of the starfish larva allowed Metchnikoff to observe the remarkable behavior of these cells, which we now know as phagocytes, thus founding the science of cellular immunology.

A hundred and fifty years later, the model organism has changed, but the principles remain the same. In a recent paper in *Cell*, Tobin and colleagues [2] followed Metchnikoff's lead and injected transparent zebrafish larvae with the bacterium *Mycobacterium marinum *to identify mutants with increased susceptibility to infection. The first such mutation identified in this screen mapped to the gene encoding the zebrafish homolog of leukotreineA4 hydrolase (LTA4H), an enzyme responsible for the conversion of LTA4 to LTB4 in macrophages (a vertebrate phagocyte). Loss of LTA4 H activity causes LTA4 to be converted to lipoxin A4 (LXA4), a related compound with immunoregulatory functions important in driving the resolution of inflammation. The overactivity of such inflammation-resolving factors underlies the hypersensitivity of the mutant animals to mycobacterial infection. Remarkably, Tobin and colleagues also found a strong association between heterozygosity at certain single nucleotide polymorphisms (SNPs) in the human *LTA4 H *locus and protection against infection and mortality from tuberculosis, as well as reduced susceptibility to severe disease in those exposed to leprosy. These striking discoveries illustrate the power of the zebrafish to yield new insights into the biology of the immune system, insights that have clear implications for our understanding and treatment of human disease.

## Triggering inflammation

**Figure 1:**
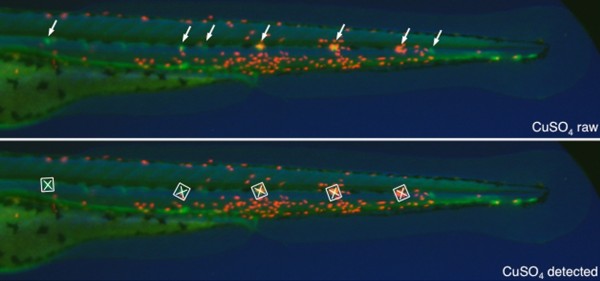
**ChIn analysis**. Compound transgenic zebrafish larvae expressing green fluorescent protein in neuromasts (arrowed in top panel) and dsRed in neutrophils. Upper panel shows recruitment of red fluorescent neutrophils to injured neuromasts in green. In the lower panel, the ChIn assay imaging scripts have identified the neuromasts (white boxes), and the amount of recruited red fluorescence (a measure of inflammation) can be assessed. Reproduced from Figure 5 in d'Alençon *et al. *[1].

Local tissue damage, such as that caused by Metchnikoff's thorn, induces the release of endogenous damage-associated molecular patterns (DAMPs), a pro-inflammatory cocktail of proteins and small-molecule mediators. In vertebrates, these signals result in the recruitment of phagocytes - neutrophils and macrophages - to the site of the lesion. Nowadays, models of the innate immune response often use sterile tissue injury as the inciting stimulus; the earliest investigation into neutrophil recruitment in the zebrafish, performed in the laboratory of Graham Lieschke, used a simple tailfin transection as the inflammatory stimulus [3]. The subsequent development of transgenic lines of zebrafish, in which innate immune cell populations are specifically labeled with fluorescent proteins and readily imaged in the transparent larva, has allowed unprecedented visualization of immune cell recruitment and inflammation resolution *in vivo *([4] and references cited therein, and [5,6]). This assay has provided the basis for genetic and pharmacological screens for novel modulators of inflammation [4,7]. But although robust and reproducible, the tailfin injury assay is time consuming and requires considerable technical skill, limiting its applicability to high-throughput screens. In the absence of efficient means of automating the process, there was a pressing need for alternative methods of initiating inflammation in the fish. d'Alençon *et al. *[1] now describe one such alternative; they show that a robust inflammatory response can be induced by localized cell death of sensory hair cells in the zebrafish lateral-line system, a process that they had previously found can be induced simply by immersion of zebrafish larvae in copper sulfate solution [8]. The response of neutrophils to copper exposure is extremely rapid: cells can be seen to start migrating towards the damaged neuromasts - discrete clusters of nerve cells regularly distributed along the length of the body - within 15 minutes of immersion of the larvae in the CuSO_4 _solution, and large accumulations of active neutrophils are apparent after 2 hours (Figure 1). The authors show that this response can be suppressed both by known anti-inflammatory drugs and by a mutation in the gene encoding the Wiskott-Aldrich syndrome protein (WASP) [9]. Moreover, by combining automated imaging with a custom software script that maps the fluorescent expression domains in the larvae, they have established a platform for high-throughput screening.

This innovation - which the authors call the chemically induced inflammation assay, or 'ChIn' assay for short - frees the neutrophil-recruitment model from the constraints imposed by manual intervention, thereby opening the door to the automation of both pharmacological and genetic screens. The zebrafish has been used for forward genetic analysis for more than 20 years, but more recently its suitability for pharmacological or 'chemical genetic' screens has become equally apparent [10]. Such whole-organism screens provide a powerful approach to the discovery of biologically active compounds that can have utility either as reagents for the dissection of biological processes or as leads for the development of therapeutics. Indeed, the first compound identified in this type of zebrafish screen is currently in phase I clinical trials to improve engraftment of transplanted cord blood stem cells, and our own experience suggests that many more will follow. The ChIn protocol will now bring screens for modulators of the innate immune response within reach of many more labs than those with established expertise in the tailfin transection assay, allowing investigators studying the molecular basis of leukocyte recruitment and inflammation resolution to add the zebrafish model to their experimental armory.

While hypothesis-driven experiments are a crucial driver of scientific knowledge, the complementary benefits of unbiased screens are clear to see. Key advances in the field of innate immunity have been made by phenotype-driven approaches and the technological innovation of d'Alençon and colleagues should accelerate both the identification of novel genes underlying the innate immune response as well as novel therapeutic approaches to its manipulation. It is worth remembering that aspirin, one of the best selling anti-inflammatory drugs of all time, and the founding member of entire classes of medicines, was similarly identified by a phenotype: the ability to reduce fever.
